# A comparative evaluation of implant stability and crestal bone loss in T-PRF-treated and conventional implants: A split-mouth clinical study

**DOI:** 10.34172/japid.4115

**Published:** 2026-08-27

**Authors:** Anuraga Tejaswi Thotimpuri, Mohan Kumar Pasupuleti, Gautami Subhadra Penmetsa, Satyanarayana Raju Manthena, Naga Venkata Satya Sruthima Gottumukkala, Konathala Santosh Venkata Ramesh

**Affiliations:** ^1^Department of Periodontics and Implantology, Vishnu Dental College, Bhimavaram, India; ^2^Department of Prosthodontics, Vishnu Dental College, Vishnupur, Bhimavaram, West Godavari, India

**Keywords:** Crestal bone loss, Dental implants, Implant stability, Split-mouth study, Titanium platelet-rich fibrin (T-PRF)

## Abstract

**Introduction:**

This study aimed to compare implant stability and crestal bone loss between titanium platelet-rich fibrin (T-PRF)-treated implants and conventional implants.

**Methods:**

A prospective, randomized, split-mouth clinical trial was conducted in 10 healthy patients, with 20 implants per mouth (test and control). Each patient received one T-PRF-treated implant and one conventional implant. Implant stability was assessed using implant stability quotient (ISQ) at placement and at 3 months. Crestal bone loss was evaluated radiographically at 3 and 6 months. Soft tissue parameters were assessed at follow-up visits.

**Results:**

The T-PRF group showed higher ISQ values and reduced bone loss, with trends favoring the intervention at the three-month follow-up compared with the control group. Bone loss at three and six months was lower around anterior implants in the T-PRF group than in the control group. At this time, T-PRF may help improve soft tissue measurements; however, the measured values did not differ significantly from those of the control groups. Due to limitations such as a small sample size, a relatively short follow-up period, and varying baseline crestal bone levels, these findings should be interpreted with caution.

**Conclusion:**

Due to the restrictions of this split-mouth study, T-PRF-treated sites showed a trend towards increased early implant stability and decreased crestal bone loss compared to control sites. However, because of the small sample size and short follow-up period, conclusive evidence cannot be drawn about the clinical superiority of T-PRF. Further appropriately powered randomized controlled trials with prolonged follow-up periods are necessary to determine the long-term clinical efficacy of T-PRF in implant therapy.

## Graphical Abstract

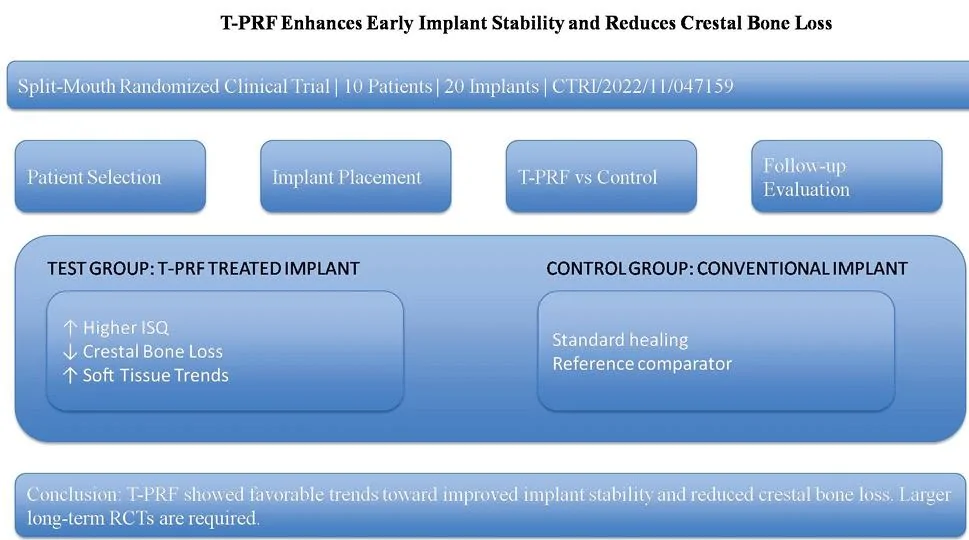

## Introduction

 Dental implants are highly successful, functionally and aesthetically pleasing solutions for tooth loss, and as such, their use has become increasingly common. Implants achieve long-term success through osseointegration, and that process depends on biological, biomechanical, and patient-related factors.^1‒3^

 To improve osseointegration, several enhancement methods have been studied, including implant surface modifications and the use of adjunct biologically active materials, such as platelet concentrates. Platelet-rich fibrin (PRF) is a second-generation platelet concentrate that can act as an organic scaffold releasing growth factors (PDGF, TGF-β, and VEGF) to promote tissue regeneration and angiogenesis.^3‒5^

 Although conventional PRF has become popular as an autologous regenerative biomaterial, it has several drawbacks. When made in regular glass tubes the fibrin matrix breaks down fairly and quickly, leading to a short time for scaffold function.^6^ Furthermore, the release of growth factors is not steady, with many bioactive molecules being released soon after surgery. This fast degradation and burst release may hinder the extended biological stimulation necessary for effective tissue regeneration.^7‒9^ In 2014, Tunali et al.^10^ introduced titanium platelet-rich fibrin (T-PRF) to overcome these issues. They used a titanium tube to create a denser and longer-lasting fibrin matrix with better properties.^10^

 Preliminary study data suggest that T-PRF may improve peri-implant healing and minimize marginal bone loss; however, there remains a lack of large, long-term studies and limited methodological consistency. Direct head-to-head comparison studies between T-PRF and traditional implant protocols with controlled split-mouth designs have been limited.^9‒11^

 Therefore, the present study quantitatively compared implant stability and crestal bone loss between T-PRF-treated and conventional implants using resonance frequency analysis (RFA) and radiographic evaluation.

## Methods

###  Study Design and Ethical Approval

 The Department of Periodontics and Implantology at Vishnu Dental College conducted this randomized, split-mouth clinical trial in accordance with the CONSORT guidelines. Split-mouth designs reduce inter-individual variability because each patient serves as their own control, allowing meaningful comparisons with smaller samples. After obtaining signed informed consent, 10 systemically healthy participants aged 25‒45 years were included in the study. Although the calculated mean age and standard deviation suggest variability, all enrolled participants were verified to fall within the predefined age range. Authorization by the Institutional Ethics Committee (Ref. No. [IECVDC/2022/PG01/PI/IVV/16]) was obtained prior to the study, and the study was prospectively registered with the Clinical Trials Registry of India (CTRI/2022/11/047159).

###  Sample Size and Statistical Considerations

 The a priori sample size calculation indicated a requirement of 17 participants per group; however, only 10 participants were included due to feasibility constraints. Consequently, the study was underpowered to reliably detect inter-group differences, particularly for moderate or small effect sizes. Although a split-mouth design reduces inter-individual variability, it does not compensate for insufficient statistical power in hypothesis testing.

 Furthermore, the assumed effect size (Cohen’s d = 1.0) used for sample size estimation was relatively large, potentially overestimating the expected treatment effect and thereby underestimating the required sample size. This increased the risk of type II error and limited the robustness of statistical inferences.

 Given these limitations, the findings of this study should be interpreted as exploratory rather than confirmatory. While *P*-values have been reported, they should not be considered definitive evidence of treatment effects in this underpowered context. Greater emphasis should instead be placed on the magnitude and direction of observed effects, including effect sizes and corresponding confidence intervals, which provide more meaningful insights into potential clinical relevance (Figure 1).

**Figure 1:**
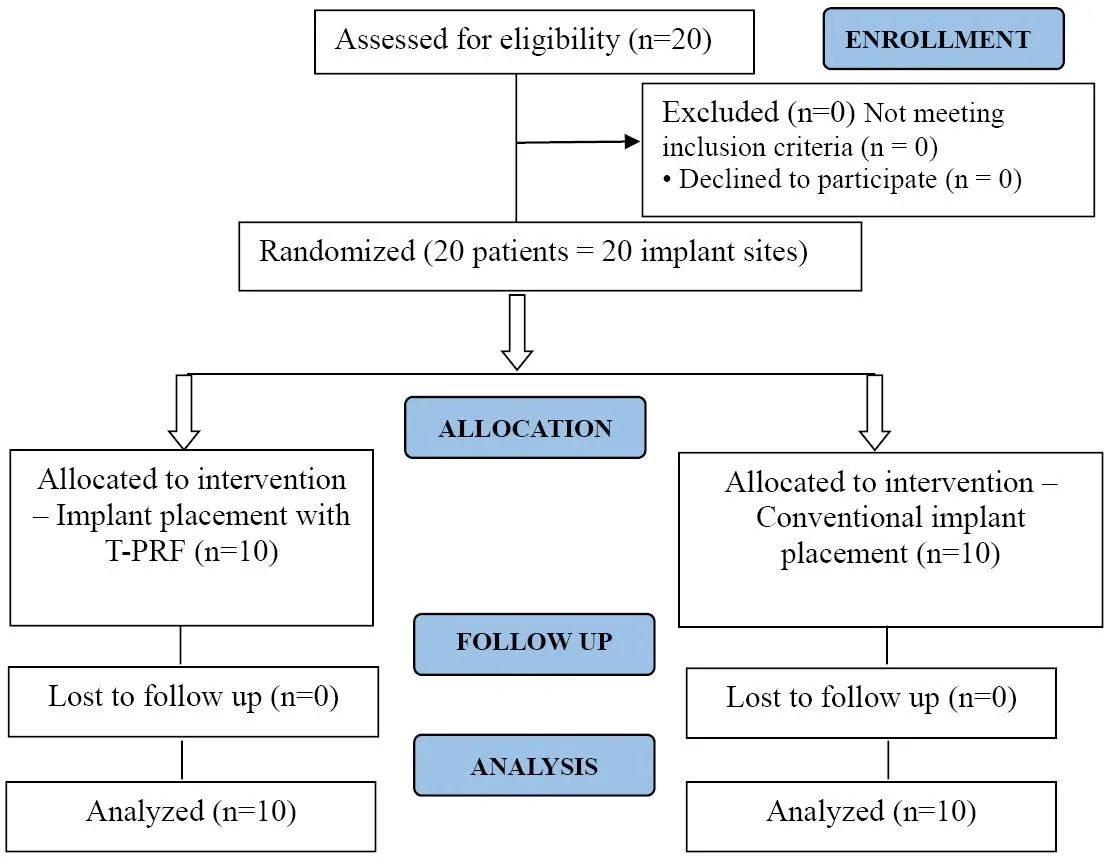
CONSORT 2010 flow diagram depicting patient enrolment, randomization, allocation, follow-up, and analysis in a randomized split-mouth clinical trial comparing T-PRF-coated and conventional dental implants. No patients or implant sites were lost to follow-up, and all randomized sites were included in the final analysis

###  Inclusion Criteria

 Patients aged 25–45 years with missing mandibular posterior teeth

 Adequate bone height and width for implant placement

 Good oral hygiene and willingness to comply with follow-up visits

###  Exclusion Criteria

 Systemic diseases affecting bone metabolism (e.g., uncontrolled diabetes)

 Patients on anticoagulants, bisphosphonates, or statins

 Pregnant or lactating women

 Smokers or patients undergoing chemotherapy or hormone therapy

###  Randomization and Allocation

 Eligible patients were randomly assigned to receive T-PRF-treated and conventional implants using computerized randomization. Each patient received two implants (DIO, 4.5 × 10 mm), one on each side of the mandible: the test site was treated with T-PRF, and the contralateral site received a conventional implant.

###  Preoperative Assessment

 An extensive case history, intraoral photographs, and diagnostic impressions were taken. Radiographic evaluation included intraoral periapical radiographs (IOPAs) and cone-beam computed tomography (CBCT) scans. Complete blood investigations were completed to ensure there were no systemic issues for surgery.

###  Soft Tissue Assessment

 In addition to measuring hard tissue parameters, the soft tissues were evaluated using a variety of established methods at both baseline and follow-up visits. The thickness of the gingival tissue at the mid-buccal site was assessed using a transgingival probe, following a standardized protocol to ensure accuracy. The distance from the free gingival margin (FGM) to the cementoenamel junction (CEJ) was measured using a calibrated periodontal probe to assess the gingival margin. Using the papilla index score, the gingival index (GI), and the plaque index (PI), the interdental papilla fill was measured and used to evaluate gingival inflammation and the presence or absence of plaque, respectively. Bleeding on probing (BOP) was assessed by recording whether it occurred within 15 seconds of gentle probing. All measurements were taken by a single experienced examiner; thus, inter-examiner variability would not influence outcomes.

###  Preparation of Titanium Platelet-Rich Fibrin (T-PRF)

 Ten mL of venous blood was collected from each participant using sterile titanium tubes without anticoagulants. Blood samples were centrifuged immediately after collection, within 1–2 minutes, to prevent premature clotting. Centrifugation was performed at 3000 rpm for 15 minutes, corresponding to ~700–800 × g (relative centrifugal force), depending on the rotor radius of the centrifuge used.

 The titanium tubes (medical-grade titanium; T-Lab®, Izmir, Türkiye) were selected to avoid silica contamination associated with glass tubes and to promote the formation of a denser fibrin matrix. Following centrifugation, the fibrin clot formed in the upper layer was carefully retrieved, separated from the red blood cell layer, and gently compressed into a membrane for clinical application.

###  Surgical Protocol

 All surgical procedures were performed by the same experienced operator under aseptic conditions. Following local anesthesia (2% lidocaine with 1:100,000 epinephrine), a mid-crestal incision with full-thickness flap elevation was performed.

 Osteotomy sites were prepared sequentially using a standardized implant drilling protocol under copious saline irrigation (duplicate sentence removed). Commercially available implants (DIO Implant Co., Busan, South Korea; 4.5 × 10 mm) were placed according to the manufacturer’s instructions. Insertion torque values (Ncm) were recorded at the time of implant placement to assess primary stability.

 Control site: Conventional implant placement without T-PRF application (Figure 2).

**Figure 2:**
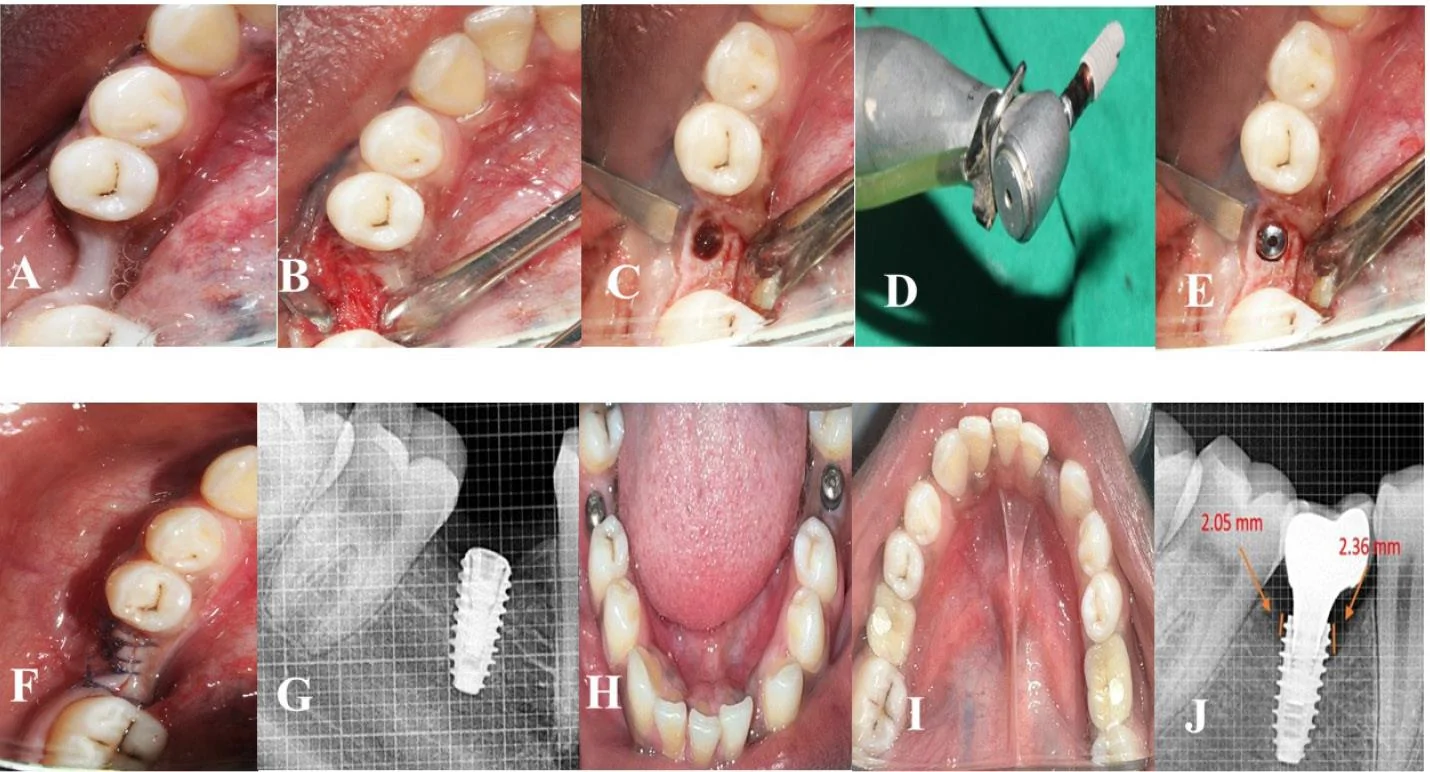
Control group: Step-by-step procedure of conventional implant placement. A) Preoperative site. B) Flap elevation. C) Osteotomy site. D) Implant to be placed. E) Cover screw placed. F) Sutures placed. G) Postoperative RVG. H) Healing abutment placed. I) Crown cementation done. J) 6-month follow-up RVG

 Test site: The osteotomy site was lined with a T-PRF membrane prior to implant placement (Figure 3).

**Figure 3:**
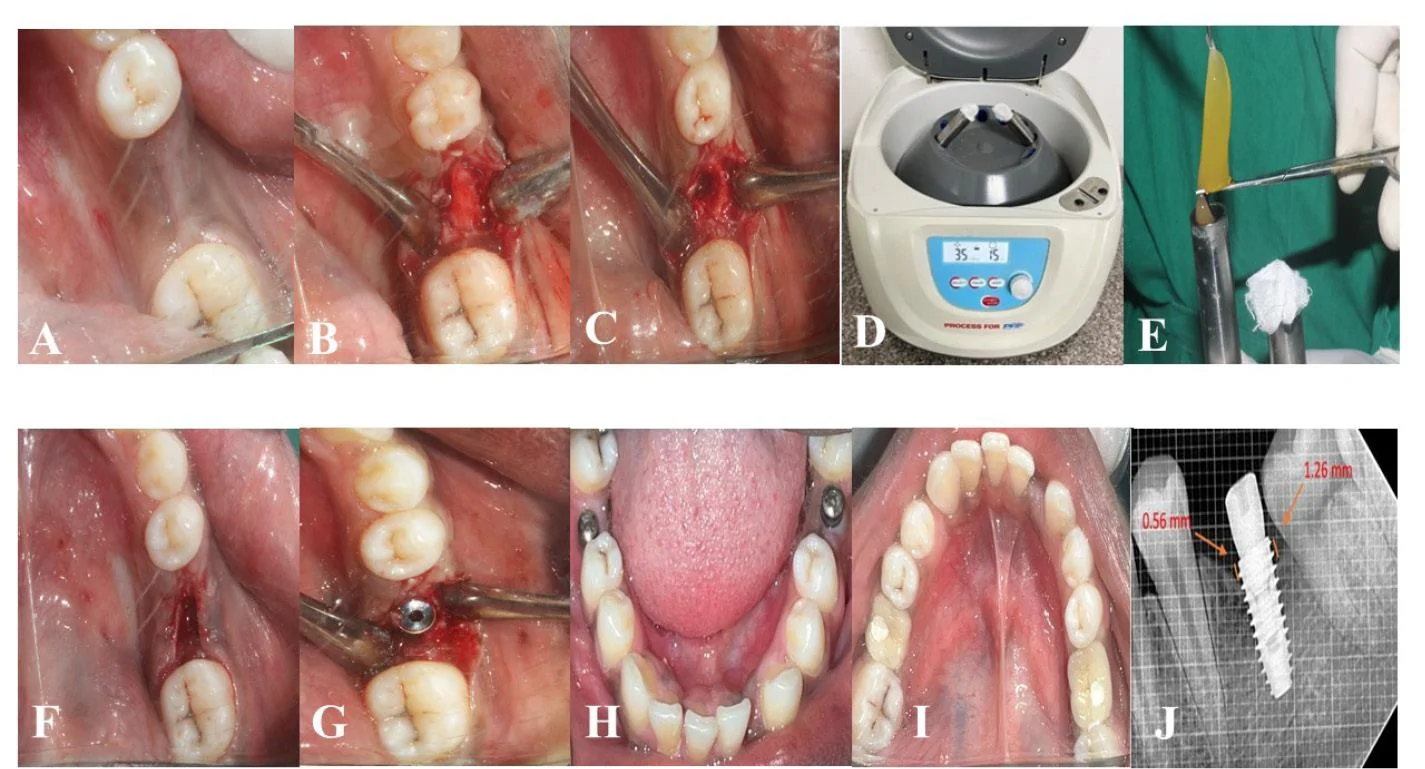
Control group: Step-by-step procedure of conventional implant placement. A) Preoperative site. B) Flap elevation. C) Osteotomy site. D) Implant to be placed. E) Cover screw placed. F) Sutures placed. G) Postoperative RVG. H) Healing abutment placed. I) Crown cementation done. J) 6-month follow-up RVG

 Implant placement was performed following the manufacturer’s recommended drilling sequence. Insertion torque values (measured in Ncm) were recorded at the time of implant placement using a calibrated torque wrench to assess primary stability at both test and control sites. All implants were placed at the crestal bone level and secured with cover screws, followed by suturing using 4-0 Vicryl.

 Each implant was placed at the level of the bone cortex, and a cover screw was placed. The implants were secured with interrupted sutures (4-0 Vicryl) placed in a simple way. Patients received antibiotics (amoxicillin) for 5 days at 500 mg tid, anti-inflammatories (diclofenac sodium) for 3 days at 50 mg bid, and proton pump inhibitors (pantoprazole) for 3 days at 40 mg od after their surgeries.

 To reduce the risk of early dental implant failure and postoperative infection, we administered antibiotics prophylactically. Each patient received 1 g of amoxicillin as a single dose one hour before surgery, and then continued on amoxicillin 500 mg three times per day for the following five days. This antibiotic protocol is supported by evidence suggesting that antibiotic prophylaxis may reduce early dental implant failure rates in dental implant surgeries, specifically those involving flap elevation and implant placement (according to a study by Romandini et al,^12^ in 2019). The postoperative antimicrobial regimen we used is representative of the institutional protocol followed for all patients included in the study.^12^

###  Postoperative Care and Follow-up

 Patients were asked to maintain oral hygiene and avoid any mechanical trauma to the surgical site. Sutures were removed at one week. All the patients were followed up at 3 and 6 months. Any postoperative complications (infection, implant failure, sensory changes) were recorded.

###  Radiographic Assessment of Crestal Bone Levels

 Standardized RVGs using the long-coned vertical angle technique were obtained from all patients at baseline (when the implants were placed), three months after placement, and six months after placement. All the patients had a custom-made acrylic holding device or a holding device to help ensure the sensor was positioned properly relative to the implants and adjacent teeth, both before the implants were placed and during all follow-up periods. After this was achieved, we continued to use the same holding device and alignment devices to ensure reproducibility of both vertical and horizontal angulation.

 Crestal bone levels were measured digitally using image analysis software, and linear measurements were made from the top of the implant to the first point of contact of the bone–implant interface at the mesial and distal sides of the implant. Once the linear measurements had been taken, we converted them from pixels to millimeters by calibrating the image using the actual implant thread pitch (i.e., the distance between two adjacent threads).

 Radiographic measurements were performed by a single blinded examiner using standardized positioning and image analysis software. To assess intra-examiner reliability, 20% of the images were randomly re-measured after a two-week interval. A blinded examiner took the above measurements prior to data collection. To ensure intra-examiner reliability, the examiner completed a calibration exercise using a subset of radiographs before collecting the data. After the examiner measured the study population’s radiographs, 20% of them were re-measured at a two-week interval to complete the ICC statistics. All calculations for these ICCs were performed using the two-week-interval data shape.

###  Outcome Measures

####  Implant Stability

 Implant stability quotient (ISQ) was estimated at baseline (immediately after insertion) and at 3 months with a resonance frequency analyzer (RFA).

####  Crestal Bone Level Changes

 Standardized digital radiographs (RVG) were taken at baseline, 3 months, and 6 months. Mesial and distal crestal bone levels were measured using a fixed implant reference point with image analysis software.

###  Statistical Analysis

 Given the split-mouth design, all inter-site comparisons (T-PRF vs. control) were performed using paired statistical methods, as each participant served as their own control. Continuous data were presented as mean ± standard deviation along with mean differences and effect sizes (Cohen’s d), with confidence intervals reported where appropriate.

 Due to the small sample size (n = 10), formal normality testing using Shapiro–Wilk and homogeneity testing using Levene’s test were not emphasized, as these tests have limited power in small samples. Where appropriate, non-parametric alternatives (Wilcoxon signed-rank test) were considered to support the robustness of findings.

 Given the exploratory nature of this pilot study and its limited statistical power, inferential statistics were interpreted with caution, with greater emphasis on the magnitude and direction of observed effects rather than on statistical significance.

 Analyses were supplemented by change-from-baseline comparisons to account for initial differences between sites.

 Independent t-tests and baseline-adjusted models (e.g., ANCOVA) were not applied due to the small sample size and paired study design, which may limit the stability of such analyses. Adjustments for multiple comparisons (e.g., Bonferroni correction) were not applied, as they may further reduce statistical power in this exploratory context.

## Results

 This randomized controlled split-mouth clinical trial included 10 systemically healthy patients (6 males, 4 females) with 20 implants placed bilaterally in the mandibular posterior region. The mean age was 30.0 ± 9.46 years for males and 40.5 ± 4.2 years for females (Table 1).

**Table 1:** Descriptive statistics for age and gender

**Group**	**Gender**	**N**	**Mean**	**SD**
Test	Male	6	30.0000	9.46573
	Female	4	40.5000	4.20317
Control	Male	6	30.0000	9.46573
	Female	4	40.5000	4.20317

 Each patient received one T-PRF-coated implant (test) and one conventional non-coated implant (control), with site allocation determined at random using a computerized randomization scheme. While operator blinding was not feasible due to the visible preparation and application of T-PRF, patients remained blinded throughout the study. The evaluators who conducted the clinical and radiographic evaluations were blinded to the patient’s group assignment. No biological or mechanical complications were observed in any implant by the end of the follow-up period, and all the patients maintained good compliance with the treatment and demonstrated good oral hygiene at baseline and throughout the 3- and 6-month evaluations.

###  Soft Tissue Parameters

 Comparing the two groups for soft tissue parameters revealed differences at 3 and 6 months. The average GI score around the T-PRF implants was 0, whereas the control group had an average of 1 (indicating mild gingival inflammation). The papilla index (Jemt Index) score was higher in the T-PRF group (mean score: 3) compared to the control group (mean score: 2), suggesting improved papillary fill in the test group. However, given the limited sample size, this observation should be interpreted cautiously. The T-PRF group’s mean gingival thickness was significantly greater than that of the control group (2.5 mm vs. 1.8 mm). The mean gingival margin position was 1.0 mm in the test group and 1.5 mm in the control group. The mean probing depths were 2.0 mm in the T-PRF and 2.5 mm in the control groups. No bleeding on probing was observed at the sites tested for T-PRF (0%), whereas mean bleeding on probing was observed at the control sites (30%). Although the T-PRF group showed numerically improved outcomes compared with the control group, the observed differences were not statistically significant, possibly reflecting the limited sample size and statistical power of this pilot study.

 Soft tissue parameters numerically favored the T-PRF group, including lower gingival inflammation, reduced probing depth, and greater gingival thickness. The observed differences were small to moderate in magnitude; however, confidence intervals were wide and included the possibility of no difference, reflecting imprecision due to the limited sample size. These findings should be interpreted as exploratory (Table 2).

**Table 2:** Comparison of soft tissue parameters between T-PRF and control groups

**Parameter**	**T-PRF Group**	**Control Group**	**Mean Difference (Test – Control)**	**Interpretation**
Gingival index (GI)	0	1	−1.0	Lower inflammation in T-PRF
Papilla index (Jemt)	3	2	+1.0	Better papillary fill in T-PRF
Gingival thickness (mm)	2.5	1.8	+0.7 mm	Greater tissue thickness in T-PRF
Gingival margin position (mm)	1.0	1.5	−0.5 mm	Slightly better marginal position
Probing depth (mm)	2.0	2.5	−0.5 mm	Shallower pockets in T-PRF
Bleeding on probing (%)	0%	30%	−30%	Reduced bleeding in T-PRF

###  Implant Stability Analysis

 Implant stability increased in both groups over time. The T-PRF group demonstrated higher mean ISQ values at baseline (81.40 ± 3.06) and at 3 months (89.90 ± 4.33) compared to the control group (74.30 ± 8.56 and 83.00 ± 6.11, respectively). The observed inter-group differences suggest a potentially meaningful effect favoring T-PRF. The magnitude of this difference was large, according to effect size estimates. However, due to the limited sample size, these findings should be interpreted with caution and treated as exploratory (Table 3).

**Table 3:** Comparison of implant stability in the test and control group at baseline and 3 months

**Time**	**Group**	**ISQ mean (mm)**	**SD**	**SD error**	**t-value**	***P*** **-value**
Baseline	Test (Group A: T-PRF)	81.4000	3.06232	.96839	2.469	0.02
	Control (Group B: conventional)	74.3000	8.56414	2.70822		
3 months	Test (Group A: T-PRF)	89.9000	4.33205	1.36991	2.913	0.01
	Control (Group B: conventional)	83.0000	6.11010	1.93218		

 Implant stability (ISQ) was measured using resonance frequency analysis. To assess intra-examiner reliability, a subset of measurements was repeated after a defined interval, and intraclass correlation coefficients (ICC) were calculated.

 Both groups demonstrated an increase in ISQ values from baseline to 3 months, suggesting improved implant stability over time (Figure 4). The magnitude of change appeared comparable between groups, although precise comparison is limited by baseline imbalance and small sample size.

**Figure 4:**
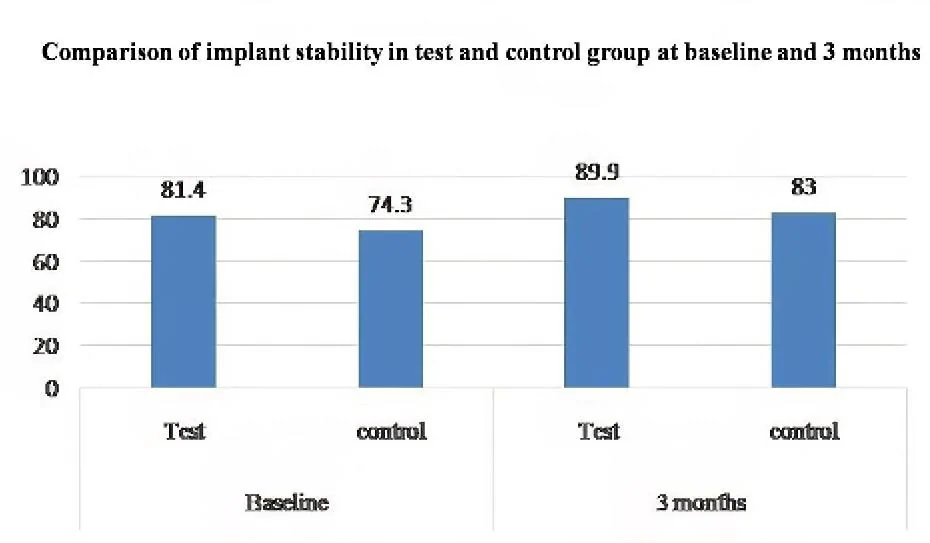
Comparison of implant stability in the test and control groups at 3 and 6 months

###  Radiographic Outcomes

 Using a custom sensor holder, RVG images were taken of periapical areas of interest using long-cone paralleling techniques. Crestal bone levels were measured digitally from the implant shoulder to the first points of contact between coronal bone and the implant on the mesial and distal surfaces. To obtain these measurements, the known pitch of the implant threads was used to calibrate the position. A blinded evaluator made radiographic measurements after being calibrated on a variety of radiographs before data collection began. To evaluate intra-evaluator reliability, 20% of the radiographic images (randomly selected from the population) were remeasured by the same evaluator two weeks after their initial measurement. The ICCs obtained were 0.91 for mesial and 0.89 for distal bone-level measurements, indicating very good repeatability of the evaluator’s measurements.

 At 3 months, lower crestal bone loss was observed in the T-PRF group compared to controls (mesial: 0.93 ± 0.10 mm vs. 1.09 ± 0.08 mm; distal: 0.82 ± 0.15 mm vs. 1.03 ± 0.07 mm). The mean differences were −0.16 mm (95% CI: −0.22 to −0.09) at the mesial surface and −0.21 mm (95% CI: −0.32 to −0.11) at the distal surface, indicating a consistent trend favoring T-PRF. Effect size estimates suggested a large magnitude of difference. However, given the small sample size, these results should be interpreted with caution (Table 4).

**Table 4:** Mean values of crestal bone loss between groups

**Surface**	**Time interval**	**Group**	**Mean bone loss** **(mm)**	**SD**	**SE**
Mesial	3 months	Test (T-PRF)	0.93	0.10	0.03
		Control	1.09	0.08	0.02
Mesial	6 months	Test (T-PRF)	1.40	0.26	0.08
		Control	1.75	0.25	0.08
Distal	3 months	Test (T-PRF)	0.82	0.15	0.05
		Control	1.03	0.07	0.02
Distal	6 months	Test (T-PRF)	1.22	0.17	0.05
		Control	1.69	0.23	0.07

 At 6 months, both groups exhibited increased bone loss; however, the T-PRF group continued to demonstrate lower values (mesial: 1.40 ± 0.26 mm vs. 1.75 ± 0.25 mm; distal: 1.22 ± 0.17 mm vs. 1.69 ± 0.23 mm). The mean differences (−0.35 mm mesial; −0.47 mm distal) and corresponding confidence intervals indicate a sustained trend toward better crestal bone preservation with T-PRF. The effect sizes remained large, suggesting a clinically meaningful difference, although the precision of these estimates is limited by the small sample size (Table 4 and Figure 5).

**Figure 5:**
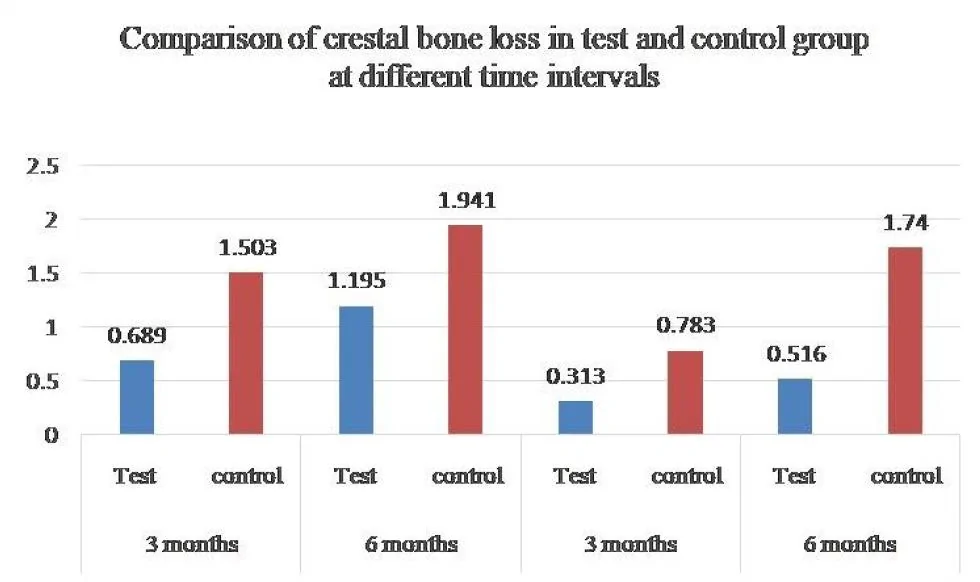
Comparison of crestal bone loss in the test and control groups at 3 and 6 months

 The application of paired-comparison analysis revealed statistically significant differences at each assessed time (three months and six months). The calculated mean difference in bone loss (mesial) at three and six months was −0.16 mm (95% CI: −0.22 to −0.09; *P* = 0.0003) and −0.35 mm (95% CI: −0.66 to −0.04; *P* = 0.03), respectively. The mean difference in bone loss (distal) at three and six months was −0.21 mm (95% CI: −0.32 to −0.11; *P* = 0.001) and −0.47 mm (95% CI: −0.72 to −0.22; *P* = 0.002), respectively. The negative mean differences confirm that crestal bone loss was significantly lower in the T-PRF group than in the control group (Table 5).

**Table 5:** Paired comparisons of crestal bone loss between groups

**Surface**	**Time**	**Mean difference (mm)**	**t value**	**95% Confidence interval**	***P*** **-value**
Mesial	3 months	-0.16	-5.56	-0.22 to -0.09	0.0003
Mesial	6 months	-0.35	-2.56	-0.66 to -0.04	0.03
Distal	3 months	-0.21	-4.69	-0.32 to -0.11	0.001
Distal	6 months	-0.47	-4.29	-0.72 to -0.22	0.002

 The effect size analyses further support these findings clinically, based on Cohen’s d values, which indicate large effect sizes at both the three- and six-month time points: 1.76 and 0.81 for the mesial surface, and 1.48 and 1.36 for the distal surface, respectively. The values indicate large effects based on how substantial the corresponding mean values are to each other. Thus, the data support the conclusion that the application of T-PRF results in statistically significant reductions in crestal bone loss; this reduction is clinically meaningful relative to the application of T-PRF (Table 6).

**Table 6:** Effect size analysis (Cohen’s d)

**Surface**	**Time**	**Cohen’s d**	**Interpretation**
Mesial	3 months	1.76	Large effect
Mesial	6 months	0.81	Large effect
Distal	3 months	1.48	Large effect
Distal	6 months	1.36	Large effect

Statistically significant differences were observed on both mesial and distal surfaces at all time points (*P* < 0.05). Effect size analysis further indicated large effect sizes, suggesting a substantial reduction in early crestal bone loss associated with T-PRF application

 The results of the intra-examiner reliability analysis indicated that radiographic measurements were highly reproducible. The intraclass correlation coefficient (ICC) for mesial bone levels was 0.91 (CI: 0.84–0.96), and 0.89 (CI: 0.81–0.95) for distal bone levels, thereby indicating that the measurements taken using the protocol were very consistent and provided evidence of the validity of the radiographic evaluation method utilized in this study (Table 7).

**Table 7:** Intra-examiner reliability of radiographic measurements

**Parameter**	**ICC**	**95% CI**	**Interpretation**
Mesial bone level	0.91	0.84–0.96	Excellent reliability
Distal bone level	0.89	0.81–0.95	Excellent reliability

Radiographic measurements showed excellent intra-examiner reliability, with intraclass correlation coefficients greater than 0.85, confirming the reproducibility

###  Summary of Findings

 Both groups exhibited a stable increase in implant stability over time; however, the T-PRF group had much higher values than those in the control group. Also, the differences between groups at the baseline were greater than those observed after the procedure; thus, the differences observed between groups may reflect an initial imbalance rather than a treatment effect.

 The mean crestal bone loss (between mesial and distal sites) following treatment was lower in the T-PRF group compared to the control group. Therefore, T-PRF treatment may be developing improved support of the peri-implant bone.

 Comparison of soft tissue parameters was also made between the two groups, with T-PRF-treated sites demonstrating less gingival inflammation, reduced probing depths, and superior papilla fill compared to controls.

 The above findings should be interpreted with caution due to the small sample size and baseline differences in implant stability and radiographic measurements, as well as the fact that radiograph reliability was determined using intraclass correlation coefficients (ICC) that were highly repeatable, and therefore, no statement concerning a lack of examiner calibration will be made for consistency in statement. In general, the results should be interpreted with caution.

## Discussion

 This randomized split-mouth clinical study provides preliminary exploratory evidence that titanium-prepared platelet-rich fibrin (T-PRF) may be associated with enhanced early implant stability and reduced crestal bone loss in the posterior mandible. The T-PRF group exhibited higher implant stability quotient (ISQ) values and lower crestal bone loss; however, these findings should be interpreted with caution due to the small sample size and baseline discrepancies between groups.^13‒15^

 Specifically, T-PRF subjects had higher baseline ISQ values (81.4 vs. 74.3), which may have impacted their follow-up ISQ values. Additionally, when assessing change from baseline, the two groups showed similar improvements in correlation with implant stability, indicating that the observed differences may be at least partially attributable to variations in the initial mechanical stability of the implants rather than a pure treatment effect. Further complicating the interpretation of radiographic endpoints were baseline losses in crestal bone level and the small overall difference between the two groups at follow-up (< 0.3 mm), thereby raising concerns about the extent to which these differences would be clinically meaningful.^16‒18^

 T-PRF is believed to have a biological basis for its effects. It releases growth factors over time and forms a fibrin matrix that aids in the formation of vessels, thereby increasing the speed of healing. Although these mechanisms of action are pharmacological, the lack of histological or molecular analyses in this study leaves their interpretation purely speculative.^19‒21^ Although a few studies suggest that the use of T-PRF has positive effects on the healing of soft tissue and the preservation of bone following extraction, the quality of the literature is relatively poor. Because this study was poorly designed, it does not provide confirmatory or definitive evidence for the use of T-PRF for the treatment of soft tissue and bone.^22‒24^

 Excellent intra-examiner reliability was observed for radiographic measurements (ICC = 0.91 for mesial and 0.89 for distal bone levels), indicating high measurement consistency and supporting the reliability of the reported crestal bone loss outcomes.

###  Clinical Significance of the Study

 While numerical differences favored the T-PRF group, the absolute magnitude of these changes was small and may not represent a clinically meaningful improvement. In implant dentistry, differences of this scale may fall within acceptable physiological limits and may not influence long-term treatment outcomes. Therefore, the findings, although suggestive, should not be overinterpreted in terms of clinical benefit.

###  Limitations of the Study

 This study had a number of limitations. One significant limitation was that the sample size was very small (n = 10); therefore, even though a split-mouth design was used, it was insufficient to provide our study with enough power to detect small but possibly clinically meaningful differences between the groups.

 There were baseline differences in both implant stability (ISQ) and crestal bone levels between the test and control sites prior to the start of the study; this limited the researcher’s ability to draw causal conclusions about the effect of T-PRF. It is possible that these baseline differences influenced the outcomes observed in our study, regardless of whether the patients received an intervention.

 Lastly, the short follow-up period did not allow for long-term evaluation of success, survival, stability of the implants, or healing processes; therefore, the findings of this study should be viewed as exploratory, and further well-designed, adequately powered RCTs with lengthy follow-up and standardized measurement protocols are necessary to confirm our findings.

###  Clinical Implications

 Despite the limitations of this pilot study, T-PRF could be an adjunct for implant therapy using your own blood. There is some evidence that it provides better initial implant stability and conserves the crestal bone. These results should not be viewed as unequivocal proof of clinical superiority due to the small sample size, baseline imbalances, and short follow-up period.

 Although T-PRF can be prepared quite easily from your blood without having to have anything else added to it, when it is being used clinically, the execution of the operator is likely to have an effect on how T-PRF performs, e.g., centrifuge protocol, when it was prepared relative to use, and how it was handled, all of which will introduce variation between each T-PRF sample tested.

 Additionally, although it is generally considered cost-effective, the need for specific equipment and standardized protocols may limit feasibility across clinical settings.

 Therefore, T-PRF should be regarded as a promising but investigational adjunct, and further well-designed, adequately powered clinical trials are required before routine clinical adoption can be recommended.

###  Future Research Recommendations

 As a future direction for studies, larger sample sizes with predetermined power calculations, standardized imaging protocols, longer follow-up duration, and inclusion of systemic factors (diabetes, smoking, etc.) as parameters would help further support T-PRF efficacy. The registration of the trial protocol (ClinicalTrials.gov) and documenting adverse or unintended events could help future authors increase data transparency. Finally, how T-PRF can be standardized, scaled, and used in an economically justified way in routine implantology practices is an important future direction.

## Conclusion

 Within the limitations of this pilot split-mouth clinical study, T-PRF-treated sites showed a numerical trend toward greater early implant stability and reduced crestal bone loss compared with control sites during the early healing phase. However, these observations should be interpreted with caution due to the small sample size, limited statistical power, baseline imbalances between groups, and the absence of formal calibration of radiographic examiners, all of which limit the ability to draw causal inferences.

 Therefore, the findings of this study should be considered exploratory and applicable only to early healing outcomes. Further well-designed, adequately powered randomized controlled trials with longer follow-up and standardized measurement protocols are required to determine the true clinical benefit of T-PRF in implant therapy.

## Competing Interests

 The authors declare no potential conflicts of interest with respect to the research, authorship, and/or publication of this article.

## Data Availability

 The data that support the findings of this study are available upon request from the corresponding author.

## Ethical Approval

 The Institutional Ethics Committee examined and approved the study protocol with reference number IECVDC/2022/PG01/PI/IVV/16. The trial was then registered in CTRI with reference number CTRI/2022/11/047159.
